# Pan-Genome Analysis of *Vibrio cholerae* and *Vibrio metschnikovii* Strains Isolated From Migratory Birds at Dali Nouer Lake in Chifeng, China

**DOI:** 10.3389/fvets.2021.638820

**Published:** 2021-05-31

**Authors:** Lin Zheng, Ling-Wei Zhu, Jie Jing, Jia-yao Guan, Ge-Jin Lu, Lin-Hong Xie, Xue Ji, Dong Chu, Yang Sun, Ping Chen, Xue-Jun Guo

**Affiliations:** ^1^School of Food and Engineering, Jilin Agricultural University, Changchun, China; ^2^The Key Laboratory of Jilin Province for Zoonosis Prevention and Control, Changchun, China; ^3^Wild Animal Sources and Diseases Inspection Station, National Forestry and Grassl and Bureau, Beijing, China

**Keywords:** *Vibrio cholerae*, *Vibrio metschnikovii*, comparative genomics, migratory bird, pathogenic

## Abstract

Migratory birds are recently recognized as *Vibrio* disease vectors, but may be widespread transporters of *Vibrio* strains. We isolated *Vibrio cholerae (V. cholerae)* and *Vibrio metschnikovii (V. metschnikovii)* strains from migratory bird epidemic samples from 2017 to 2018 and isolated *V. metschnikovii* from migratory bird feces in 2019 from bird samples taken from the Inner Mongolia autonomous region of China. To investigate the evolution of these two *Vibrio* species, we sequenced the genomes of 40 *V. cholerae* strains and 34 *V. metschnikovii* strains isolated from the bird samples and compared these genomes with reference strain genomes. The pan-genome of all *V. cholerae* and *V. metschnikovii* genomes was large, with strains exhibiting considerable individual differences. A total of 2,130 and 1,352 core genes were identified in the *V. cholerae* and *V. metschnikovii* genomes, respectively, while dispensable genes accounted for 16,180 and 9,178 of all genes for the two strains, respectively. All *V. cholerae* strains isolated from the migratory birds that encoded T6SS and *hlyA* were non-O1/O139 serotypes without the ability to produce CTX. These strains also lacked the ability to produce the TCP fimbriae nor the extracellular matrix protein RbmA and could not metabolize trimetlylamine oxide (TMAO). Thus, these characteristics render them unlikely to be pandemic-inducing strains. However, a *V. metschnikovii* isolate encoding the complete T6SS system was isolated for the first time. These data provide new molecular insights into the diversity of *V*. *cholerae* and *V. metschnikovii* isolates recovered from migratory birds.

## Introduction

*Vibrio* is an abundant bacterial genus in oceans and comprises numerous species including pathogenic types such as *Vibrio cholerae, Vibrio parahaemolyticus*, and *Vibrio metschnikovii*. They are often observed in high abundance in marine products. *Vibrio* are halophilic and naturally found ubiquitously in marine settings, and thus raw seafood naturally harbors these microorganisms and is the main food source responsible for gastroenteritis caused by *Vibrio* spp. (1). Cholera is caused by *V. cholerae* that carry the cholera toxin and has resulted in seven pandemics throughout human history. The seventh *V. cholerae* pandemic continues to present day, and exhibits evolved characteristics compared to previous pandemics, rendering it difficult to treat cholera disease outbreaks (2, 3). One commonality between the sixth and seventh pandemics has been observed to be associated with the CTXΦ bacteriophage that may have introduced the CTX toxin to *V. cholerae*. Nevertheless, the strains underlying the seventh pandemic did not directly evolve from those underlying the sixth pandemic (2). In contrast to *V. cholerae*, few reports have described characterizations of *V. metschnikovii*, with only some studies focused on detection and no reports on their pathogenicity.

Migratory birds have been considered potential vectors of *V. cholerae*, wherein colonization of their intestines may occur by ingesting water and marine animals infected with *V*. *cholerae* (4). Migratory birds travel over long distances and could carry pathogenic microorganisms from one region to another, spreading pathogenic microorganisms by excretion (5, 6). Therefore, migratory birds have been considered as important reservoirs of *Vibrio*, forming a fecal-food-mouth transmission route (3). Within intestinal environments, *Vibrio* are induced to evolve into treatment-resistant bacteria or pandemic strains due to stresses they are exposed to. Consequently, increased risk of bacterial spread and difficulty of bacterial source tracking occur due to the long migration distances of vector birds.

Consequently, it is important to analyze the pathogenicity of *V. cholerae* strains and whether they exhibit the potential to evolve into pandemic strains. The concept of a pan-genome was first proposed in 2005 and is a term that encompasses the sum of all genes of a species, including their core-genome, dispensable genome, and unique genome components (7). Importantly, the population-level evolutionary trends of a species can be evaluated by analyzing their pan-genome.

Migratory birds exhibiting malnutrition, wasting, diarrhea, and high mortality were observed in the Inner Mongolia region of China between 2017 and 2018. Dead birds comprised individuals from several species including *Larus ridibundus, Pluvialis squatarola, Tadorna ferrgina, Anas poecilorhyncha*, and *Aix galericulata*, in addition to other migratory birds. Moreover, 40 *V. cholerae* strains and 34 *V. metschnikovii* strains were isolated from the migratory bird epidemic materials and water environments. The *V. cholerae* strains were non-O1/O139 strains without the *ctxA/B* toxin, based on serotyping and PCR identification. To further investigate this die-off, we conducted a bacteriological examination again on migratory bird feces in Inner Mongolia, China, in 2019. However, *V. cholerae* strains were again not present in the samples, while only *V. metschnikovii* was present. In this study, we analyzed the core-/pan- genomes of *V*. *cholerae*/*V. metschnikovii* and compared them with previously sequenced strains to evaluate whether migratory birds harbor *Vibrio* spp. that have the potential to evolve into pandemic or drug-resistant strains. These analyses thus provide important data to inform the prevention and treatment of *V*. *cholerae* and *V. metschnikovii* infections and/or outbreaks.

## Materials and Methods

### Sampling

Thirty-six samples (including 10 water samples, 2 aquatic plant samples, 19 epidemic material samples, and 24 feace samples) were taken from birds in 2018 and 2019. DNA was obtained from each fecal sample by stool DNA kit, and molecular method to determine the host source of feaces (8). The visceral organ and intestinal tract samples used for this study were collected aseptically from 19 freshly dead migratory birds (not corrupt). Samples were cultivated via spread plates in triplicate and directly cultivated onto selective thiosulfate citrate bile salts sucrose (TCBS) agar plates and incubated for 24 h at 37°C. Isolates were identified using PCR amplification of *ompW, infC, ctxA, hlyA*, and *chxA* genes (9–13). The primers and conditions used for PCR amplification are described in Supplementary Table 1. In addition, *V. cholerae* isolates were subjected to O1/O139 antigen serotyping using *V. cholerae* O antisera (Tianjin Biochip Corporation). Isolates were sub-cultured at 37°C in brain heart liquid (Qingdao Haibo, China) or on CHROM agar *Vibrio* plates (CHROM, Paris, France) unless otherwise specified. Green colonies were again confirmed using serology and PCR assays (see Supplementary Table 1 for primer sequences and PCR conditions). Isolates identified as *Vibrio* were then stored in a −80°C freezer.

### Extraction of Genomic DNA and Library Construction

Genomic DNA was extracted from isolates using the Bacterial Genomic DNA Extraction Kit (Omega). Harvested DNA was then quantified using a Qubit® 2.0 Fluorometer (Thermo Scientific). A total of 1 μg of DNA per sample was used as input material for DNA library preparations. Sequencing libraries were generated using the NEBNext® Ultra™ DNA Library Prep Kit for Illumina (NEB, USA) following manufacturer's recommendations. In addition, indexing oligonucleotides were added to sequences to identify each sample. Briefly, DNA samples were fragmented by sonication to a size of 350 bp, followed by DNA fragment end-polishing, addition of poly A-tails, and ligation with full-length adaptors for Illumina sequencing, followed by additional PCR amplification. Finally, PCR products were purified (AMPure XP system), and the size distribution of the libraries was analyzed using an Agilent 2100 Bioanalyzer and quantified using real-time PCR. The whole-genomes of strains were sequenced using an Illumina NovaSeq PE150 platform at the Beijing Novogene Bioinformatics Technology Co., Ltd.

### Genome Assembly

Reads containing Illumina PCR adapters and low-quality reads were filtered from the dataset using readfq (vision 10). The remaining good quality paired reads were assembled into scaffolds using the SOAP denovo (https://sourceforge.net/projects/soapdenovo2/files/SOAPdenovo2/), SPAdes (http://cab.spbu.ru/software/spades/), and ABySS (http://www.bcgsc.ca/platform/bioinfo/software/abyss) assemblers (14–17). The filtered reads were then used to close gaps in the scaffolds using Readfq with the options: –rq1 input_1.fq, –rq2 input_2.fq,–oq1 out_1.fq,–oq2 out_2.fq,–adp1 adapter_1.lst,–adp2 adapter_2.lst,– Q QUAL, PERCENT,–C QUAL, PERCENT,–N PERCENT,–alen INT,–amis INT,–dup,–gz,–check1 read1.check,–check2 read2.check.

### Genome Feature Predictions

Genome component prediction included prediction of coding genes, repetitive sequences, non-coding RNAs, genomic islands, transposons, prophages, and clustered regularly interspaced short palindromic repeat sequences (CRISPRs). The Gene Mark program was used to identify coding genes, while interspersed repetitive sequences were predicted using the Repeat Masker (http://www.repeatmasker.org/). Tandem repeats were identified using the tandem repeats finder (TRF) program. Transfer RNA (tRNA) genes were predicted using tRNA scan-SE (18–21), while ribosomal RNA (rRNA) genes were identified using rRNAmmer (22). Small nuclear RNAs (snRNA) were predicted by BLAST searches against the Rfam database (23, 24). The Island Path-DIOMB program was used to predict genomic islands and the transposon PSI program was used to identify transposons based on the homologous blast method (25). Lastly, PHAST was used to predict prophages (http://phast.wishartlab.com/), and CRISPR Finder was used to identify CRISPRs (26, 27).

### Gene Function Prediction

Four databases were used to predict gene functions, including the Gene Ontology (GO), Kyoto Encyclopedia of Genes and Genomes (KEGG), Clusters of Orthologous Groups (COG) (28), and non-redundant (NR) protein databases (29–32). In addition, whole-genome BLAST searches were performed against the above-mentioned four databases using an *E*-value threshold of <1e-5 and a minimum alignment length percentage >40% as criteria (33). Secretory proteins were predicted using the Signal P database, and the prediction of Type I-VII proteins secreted by pathogenic bacteria was based on the EffectiveT3software program (34, 35). To evaluate pathogenic factors, pathogenicity, and drug resistance analyses were also conducted using the Virulence Factors of Pathogenic Bacteria (VFDB) and Antibiotic Resistance Genes Database (ARDB) databases (36, 37).

### Comparative Genomic Analyses

Comparative genomic analyses were conducted including analysis of genomic synteny, the distribution of core- and strain-specific genes, and phylogenetic analysis of gene families. Genomic alignment between the sample and reference genomes was performed using the MUMmer and LASTZ tools while including 41 *V. cholerae* strains and 4 *V. metschnikovii* strains as reference genomes (see Supplementary Table 2 for additional details) (38). The analysis of genomic synteny was based on alignment results. Core- and strain-specific genes were identified based on rapid clustering of similar proteins with the CD-HIT program while specifying a threshold of 50% pairwise identity and a 70% length difference cutoff. A Venn diagram was then used to show the relationships of core- and strain-specific genes among samples. The MUSCLE software program was used to align multiple single-copy core-encoded proteins identified by the core-/pan-genome analysis. The aligned sequences were then subjected to phylogenetic analysis using the TreeBeST program, a neighbor-joining tree reconstruction algorithm, and 1,000 bootstrap replicates.

Multilocus gene sequence typing was conducted based on sequence analysis of eight housekeeping genes (*adk, gyrB, mdh, metE, pgm, pntA, purM*, and *pyrC*) using previously described PubMLST protocols (https://pubmlst.org/databases.shtml). The nucleotide sequences for each locus were analyzed with the BioNumerics software program (version 7.6; Applied Maths, Belgium) and compared against published sequences on the PubMLST website. Sequence types (STs) were then determined on the basis of the eight locus allelic profiles.

### Nucleotide Sequence

#### Accession Numbers

The 74 sequences of *V. cholerae* and *V*. *metschnikovii* were submitted to FigShare under the public doi: 10.6084/m9.figshare.14417870. And C16-2-29, M13F, M9D, M21D, M28D, and M29D uploaded Genbank database at the same time, accession number GCA_014281135.1, GCA_014305065.1, GCA_014267955.1, GCA_014267965.1, GCA_014305075.1 and GCA_014305185.1, respectively.

## Results

### Genome Sequencing

A total of 40 *V. cholerae* and 34 *V. Metschnikovii* strains were identified in this study using PCR identification and comparison of gene sequences against the non-redundant (NR) protein database. All of the strains lacked the *ctxA, tcpA*, and *chxA* genes, and were also non-O1/O139 serotypes by PCR. All isolates were completely turbid after dripping *V. cholerae* O antisera and no agglutination occurred, the result was the consistent as the PCR, and it was identified as non-O1/O139 *V. cholerae* (Table 1).

**Table 1:** Characteristics of *Vibrio cholerae* and *Vibrio metschnikovii* isolates identified in migratory birds.

**Isolate name**	**Sampling date**	**Species identification**	**Host**	**Sample location**
C1F_2	2018.8.23	*V. cholerae*	*Larus ridibundus* (lung)	Chifeng, Inner Mongolia, China
C1C_1	2018.8.23	*V. cholerae*	*Larus ridibundus* (intestines)	Chifeng, Inner Mongolia, China
C1S_2	2018.8.23	*V. cholerae*	*Larus ridibundus* (kidney)	Chifeng, Inner Mongolia, China
C2XS_1	2018.8.23	*V. cholerae*	*Larus ridibundus* (kidney)	Chifeng, Inner Mongolia, China
C4C_1	2018.8.23	*V. cholerae*	*Himantopus* (intestines)	Chifeng, Inner Mongolia, China
C5G	2018.8.23	*V. cholerae*	*Pochard* (liver)	Chifeng, Inner Mongolia, China
C5G_R	2018.8.23	*V. cholerae*	*Pochard* (liver)	Chifeng, Inner Mongolia, China
C7F_1	2018.8.23	*V. cholerae*	*Larus argentatus* (lung)	Chifeng, Inner Mongolia, China
C8C_1	2018.8.23	*V. cholerae*	*Tadorna ferruginea* (intestines)	Chifeng, Inner Mongolia, China
C8C_2	2018.8.23	*V. cholerae*	*Tadorna ferruginea* (intestines)	Chifeng, Inner Mongolia, China
C11G_R	2018.8.23	*V. cholerae*	*Anas zonorhyncha* (liver)	Chifeng, Inner Mongolia, China
C11C_1	2018.8.23	*V. cholerae*	*Anas zonorhyncha* (intestines)	Chifeng, Inner Mongolia, China
C11S_2	2018.8.23	*V. cholerae*	*Anas zonorhyncha* (kidney)	Chifeng, Inner Mongolia, China
C18S_a	2018.8.30	*V. cholerae*	*Tadorna ferruginea* (kidney)	Chifeng, Inner Mongolia, China
C18p_2	2018.8.30	*V. cholerae*	*Tadorna ferruginea* (spleen)	Chifeng, Inner Mongolia, China
C18F_2	2018.8.30	*V. cholerae*	*Tadorna ferruginea* (lung)	Chifeng, Inner Mongolia, China
C18x_2	2018.8.30	*V. cholerae*	*Tadorna ferruginea* (heart)	Chifeng, Inner Mongolia, China
C18s_b	2018.8.30	*V. cholerae*	*Tadorna ferruginea* (kidney)	Chifeng, Inner Mongolia, China
C18s_2	2018.8.30	*V. cholerae*	*Tadorna ferruginea* (kidney)	Chifeng, Inner Mongolia, China
C18c_3	2018.8.30	*V. cholerae*	*Tadorna ferruginea* (intestines)	Chifeng, Inner Mongolia, China
C18G_a	2018.8.30	*V. cholerae*	*Tadorna ferruginea* (liver)	Chifeng, Inner Mongolia, China
C18F_b	2018.8.30	*V. cholerae*	*Tadorna ferruginea* (lung)	Chifeng, Inner Mongolia, China
C18G_b	2018.8.30	*V. cholerae*	*Tadorna ferruginea* (liver)	Chifeng, Inner Mongolia, China
C18c_a	2018.8.30	*V. cholerae*	*Tadorna ferruginea* (intestines)	Chifeng, Inner Mongolia, China
C18x_b	2018.8.30	*V. cholerae*	*Tadorna ferruginea* (heart)	Chifeng, Inner Mongolia, China
C19p_c	2018.8.30	*V. cholerae*	*Tadorna ferruginea* (spleen)	Chifeng, Inner Mongolia, China
C19F_3	2018.8.30	*V. cholerae*	*Tadorna ferruginea* (lung)	Chifeng, Inner Mongolia, China
C19c_a	2018.8.30	*V. cholerae*	*Tadorna ferruginea* (intestines)	Chifeng, Inner Mongolia, China
C19c_B	2018.8.30	*V. cholerae*	*Tadorna ferruginea* (intestines)	Chifeng, Inner Mongolia, China
C19G_b	2018.8.30	*V. cholerae*	*Tadorna ferruginea* (liver)	Chifeng, Inner Mongolia, China
C19F_1	2018.8.30	*V. cholerae*	*Tadorna ferruginea* (lung)	Chifeng, Inner Mongolia, China
C19S_b	2018.8.30	*V. cholerae*	*Tadorna ferruginea* (kidney)	Chifeng, Inner Mongolia, China
C19x_a	2018.8.30	*V. cholerae*	*Tadorna ferruginea* (heart)	Chifeng, Inner Mongolia, China
C19c_1	2018.8.30	*V. cholerae*	*Tadorna ferruginea* (intestines)	Chifeng, Inner Mongolia, China
C19x_b	2018.8.30	*V. cholerae*	*Tadorna ferruginea* (heart)	Chifeng, Inner Mongolia, China
C19G_a	2018.8.30	*V. cholerae*	*Tadorna ferruginea* (liver)	Chifeng, Inner Mongolia, China
C19p_b	2018.8.30	*V. cholerae*	*Tadorna ferruginea* (spleen)	Chifeng, Inner Mongolia, China
C2W	2018.8.30	*V. cholerae*	Water	Chifeng, Inner Mongolia, China
C7W	2018.8.30	*V. cholerae*	Water	Chifeng, Inner Mongolia, China
C16_2_290	2017.8.4	*V. cholerae*	*Phalacrocorax*	Wuliangsuhai, Inner Mongolia, China
M4G_2	2018.8.23	*V. metschnikovii*	*Himantopus mexicanus* (liver)	Chifeng, Inner Mongolia, China
M5C_1	2018.8.23	*V. metschnikovii*	*Pochard* (intestines)	Chifeng, Inner Mongolia, China
M5C_2	2018.8.23	*V. metschnikovii*	*Pochard* (intestines)	Chifeng, Inner Mongolia, China
M7S_1	2018.8.23	*V. metschnikovii*	*Larus argentatus* (kidney)	Chifeng, Inner Mongolia, China
M7C_1	2018.8.23	*V. metschnikovii*	*Larus argentatus* (intestines)	Chifeng, Inner Mongolia, China
M9G_2	2018.8.23	*V. metschnikovii*	*Tadorna ferruginea* (liver)	Chifeng, Inner Mongolia, China
M11F_1	2018.8.23	*V. metschnikovii*	*Anas poecilorhyncha* (lung)	Chifeng, Inner Mongolia, China
M12C	2018.8.30	*V. metschnikovii*	*Pochard* (intestines)	Chifeng, Inner Mongolia, China
M13F	2018.8.30	*V. metschnikovii*	*Aix galericulata* (lung)	Chifeng, Inner Mongolia, China
M14Y	2018.8.30	*V. metschnikovii*	*Tadorna ferruginea* (pancreas)	Chifeng, Inner Mongolia, China
M14Y_1	2018.8.30	*V. metschnikovii*	*Tadorna ferruginea* (pancreas)	Chifeng, Inner Mongolia, China
M17C_1	2018.8.30	*V. metschnikovii*	Migratory birds (intestines)	Chifeng, Inner Mongolia, China
M19C_2	2018.8.30	*V. metschnikovii*	*Tadorna ferruginea* (intestines)	Chifeng, Inner Mongolia, China
M19C_6	2018.8.30	*V. metschnikovii*	*Tadorna ferruginea* (intestines)	Chifeng, Inner Mongolia, China
M1W	2018.8.30	*V. metschnikovii*	Water	Chifeng, Inner Mongolia, China
M3W	2018.8.30	*V. metschnikovii*	Water	Chifeng, Inner Mongolia, China
MH3GW	2018.8.30	*V. metschnikovii*	Water	Chifeng, Inner Mongolia, China
MNW	2018.8.30	*V. metschnikovii*	Water	Chifeng, Inner Mongolia, China
MNW_3	2018.8.30	*V. metschnikovii*	Water	Chifeng, Inner Mongolia, China
M3X	2019.7	*V. metschnikovii*	*Larus ridibundus*	Chifeng, Inner Mongolia, China
M7D	2019.7	*V. metschnikovii*	*Larus ridibundus*	Chifeng, Inner Mongolia, China
M8D	2019.7	*V. metschnikovii*	*Larus ridibundus*	Chifeng, Inner Mongolia, China
M9D	2019.7	*V. metschnikovii*	*Larus ridibundus*	Chifeng, Inner Mongolia, China
M14D	2019.7	*V. metschnikovii*	*Larus ridibundus*	Chifeng, Inner Mongolia, China
M15D	2019.7	*V. metschnikovii*	*Larus ridibundus*	Chifeng, Inner Mongolia, China
M16X	2019.7	*V. metschnikovii*	*Larus ridibundus*	Chifeng, Inner Mongolia, China
M19X	2019.7	*V. metschnikovii*	*Larus ridibundus*	Chifeng, Inner Mongolia, China
M21D	2019.7	*V. metschnikovii*	*Larus ridibundus*	Chifeng, Inner Mongolia, China
M26X	2019.7	*V. metschnikovii*	*Larus ridibundus*	Chifeng, Inner Mongolia, China
M27D	2019.7	*V. metschnikovii*	*Larus ridibundus*	Chifeng, Inner Mongolia, China
M28D	2019.7	*V. metschnikovii*	*Larus ridibundus*	Chifeng, Inner Mongolia, China
M29D	2019.7	*V. metschnikovii*	*Larus ridibundus*	Chifeng, Inner Mongolia, China
M19_1W	2019.7	*V. metschnikovii*	Water	Chifeng, Inner Mongolia, China
M19_6WC	2019.7	*V. metschnikovii*	Aquatic plants	Chifeng, Inner Mongolia, China

### Core- and Pan-Genomic Analysis

**Figure 1:**
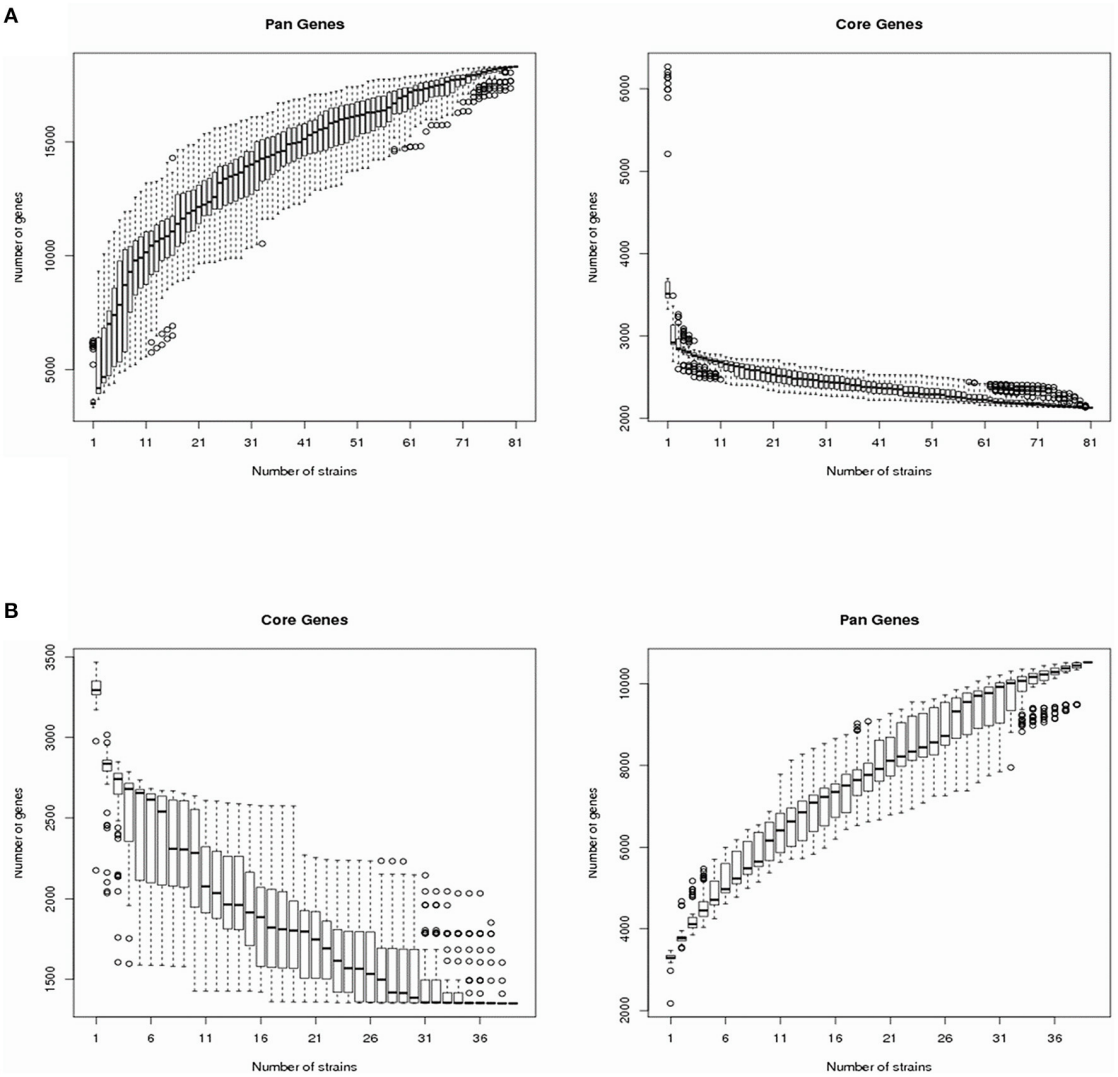
*Vibrio cholerae* and *Vibrio metschnikovii* core-/pan-genome diversity curves. **(A)** The *V. cholerae* core- and pan-genomes are shown as a function of the number of genomes included in the counts. Boxes represent one standard deviation around the median number of genes within the subsets, while the whiskers indicate two standard deviations from the median. **(B)** The *V. metschnikovii* core- and pan-genomes are shown as a function of the number of genomes included in the counts. Boxes represent one standard deviation around the median number of genes within the subsets, while the whiskers indicate two standard deviations from the median.

The *V*. *cholerae* pan-genomes were analyzed by dividing them into core and dispensable genomes. A total of 40 *V*. *cholerae* strains isolated from migratory birds were used in comparison against 41 reference *V*. *cholerae* genomes retrieved from GenBank. The number of core genes and the pan-genome sizes of the *V. cholerae* strains are shown in Figure 1A as a function of the number of genes within the genomes. The number of core genes plateaued when plotting the number of core genes against the reciprocal of the number of genomes included in the estimates. In contrast, the pan-genome size steadily increased with the addition of each additional genome, suggesting that *V*. *cholerae* exhibited a large pan-genome. Overall, a total of 18,310 pan-genes, 2,130 core-genes, and 16,180 dispensable genes were identified among all *V*. *cholerae* strain genomes (Supplementary Table 3). In this study, the dispensable genome was considered to comprise strain-specific genes. A heatmap was constructed to identify the distribution of dispensable genes among different *V*. *cholerae* strains. With the exception of the strain TSY216 genome that contained 984 dispensable genes, most other strains in the group C clade contained few dispensable genes.

To analyze the *V. metschnikovii* pan-genome, 34 *V. metschnikovii* strain genomes recovered from migratory bird isolates and four reference genomes of *V. metschnikovii* strains were used for comparative analysis. The size of the pan-genome steadily increased with the addition of each additional genome in the analysis (Figure 1B), suggesting that *V. metschnikovii* also exhibited a large pan-genome. A total of 10,530 pan-genes, 1,352 core-genes, and 9,178 dispensable genes were identified among all *V. metschnikovii* strains. Like the genomes of the *V. cholerae* strains, most *V. metschnikovii* genomes did not contain many dispensable genes, with the exception of strain M13F, whose genome contained 1,048 dispensable genes.

### Phylogenetic Analysis

**Figure 2:**
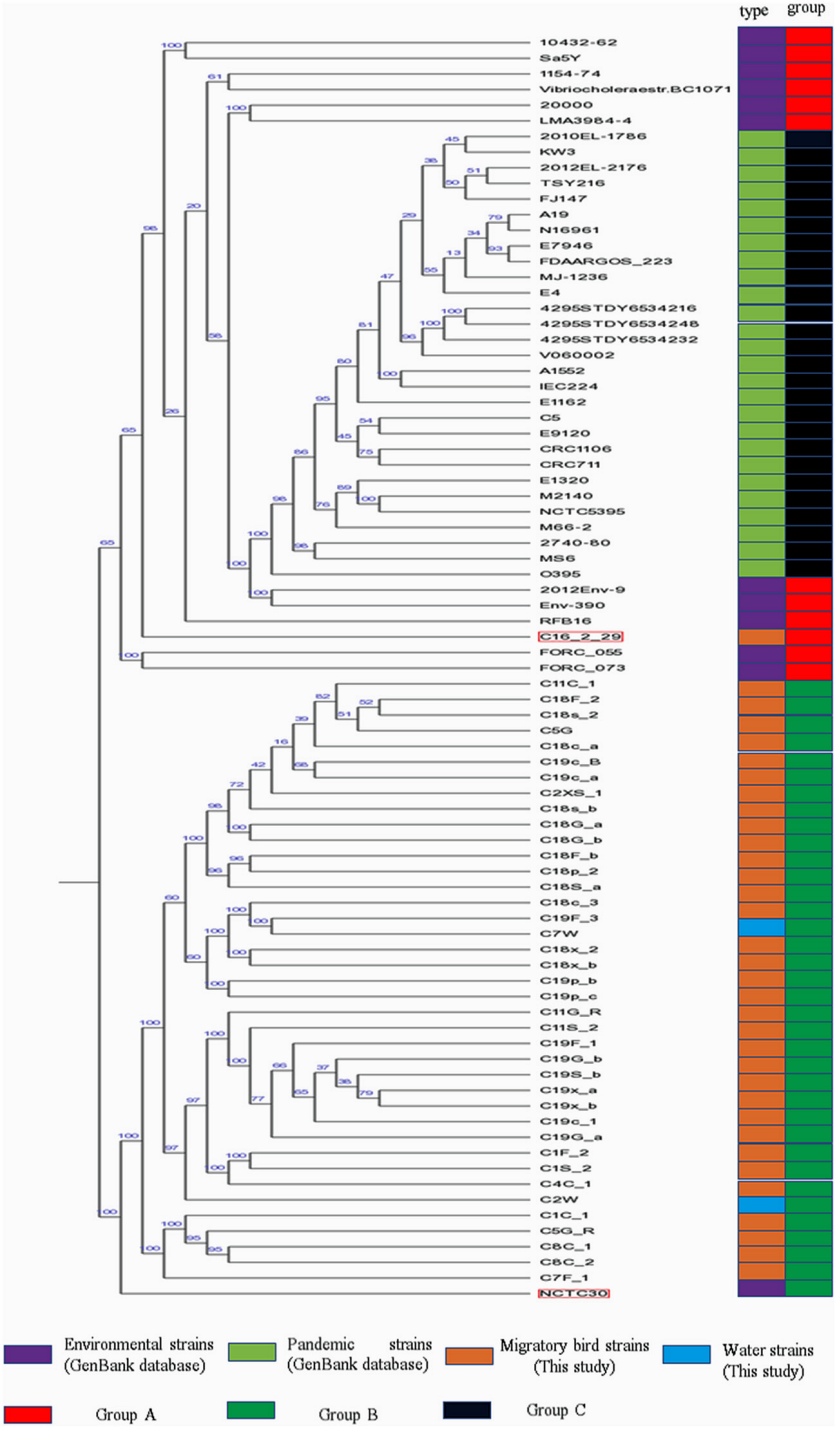
Phylogenetic analysis of *V*. *cholera* strains. Different colored blocks are used to indicate types and groups of strains on the left of phylogenetic tree. The color legend is shown below the phylogenetic tree.

Phylogenetic analysis indicated the presence of similar branching patterns, wherein the *V*. *cholerae* genomes consistently grouped into two major clades. The first clade contained *V. cholerae* strains from the GenBank reference database and one strain (C16-2-29) from our study. All of the other reference strains, with the exception of strain NCTC30, were present in this clade. In the first clade, strains harboring the cholera toxin (CTX) were divided into group C, while the others in the first clade were considered as group A. All of the strains identified in this study, with the exception of strain C16-2-29 (Wuliangsuhai, 2017), were present in the second clade, which was designated as Group B (Figure 2).

MLST analysis of the pandemic *V. cholerae* strains was conducted using eight different loci (*adk, gyrB, mdh, metE, pgm, pntA, purM*, and *pyrC*). However, only seven different loci (*adk, gyrB, mdh, metE, pntA, purM*, and *pyrC*) were used for the MLST of the environmental *V. cholerae* strains (Supplementary Table 3), since *pgm* was not detected in the environmental strain genomes. With the exception of the C16-2-29 genome, most of the housekeeping genes of *V*. *cholerae* strains from migratory birds did not exhibit homology to known genotypes in the pubMLST database. In addition, the *pgm* gene was detected in the strain C16-2-29 genome, as observed for the pandemic strain genomes. ST69 was the common sequence typing classification for the pandemic strains, while the sixth pandemic strain, O395, exhibited an ST73 sequence type. ST69 was not observed for the *V. cholerae* strains derived from environments or migratory birds.

**Figure 3:**
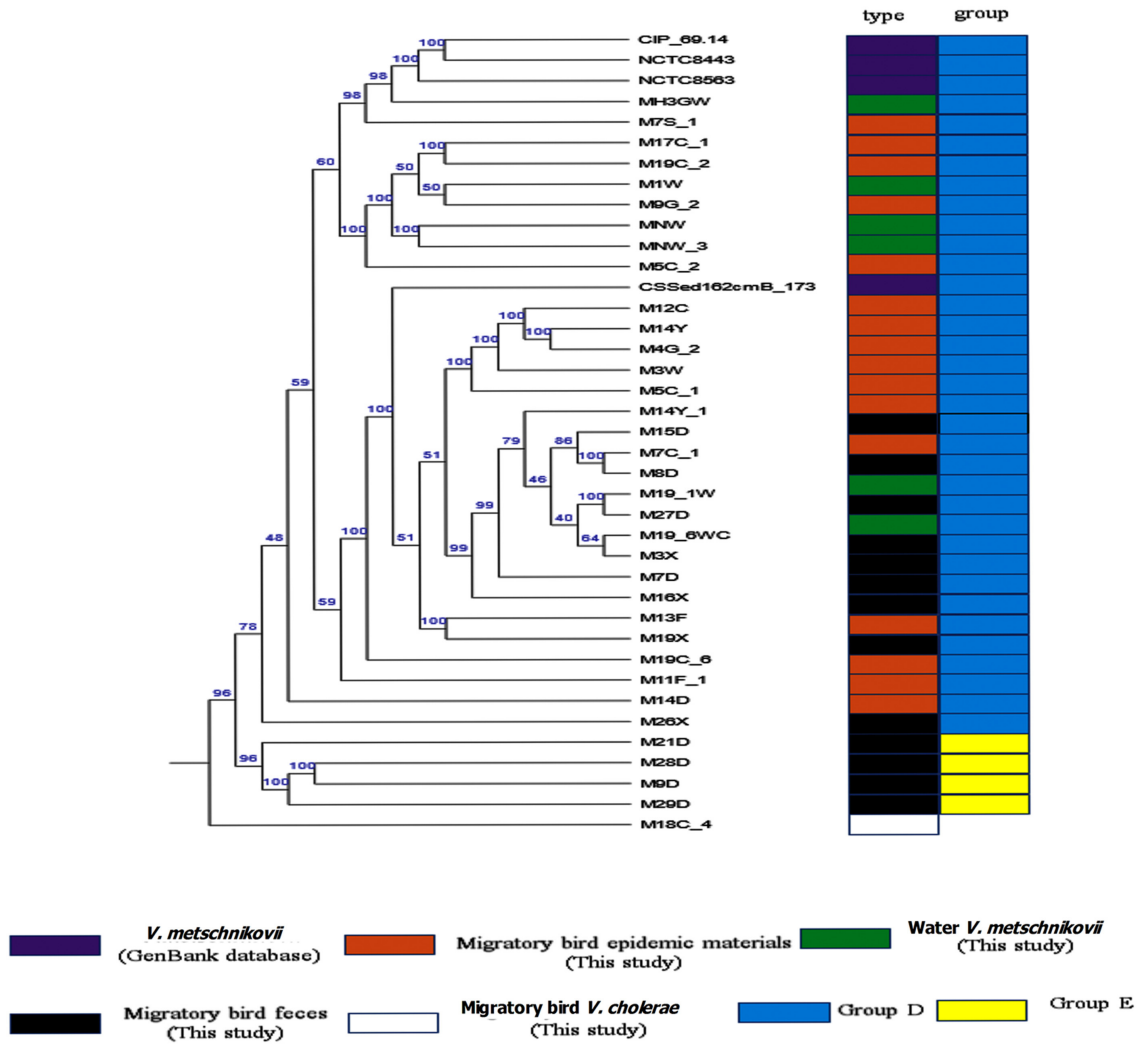
Phylogenetic analysis of *V. metschnikovii* strain genomes. Different colored blocks are used to indicate types and groups of strains on the left of phylogenetic tree. The color legend is shown below the phylogenetic tree.

Phylognetically, the *V. metschnikovii* and *V*. *cholerae* strains were genetically highly distant. The *V. metschnikovii* genomes consistently grouped into two major clades, with the first comprising *V. metschnikovii* strains from different samples and the reference genomes. This first group was designated as Group D. The second clade comprised four strains (M21D, M28D, M9D, and M29D) designated as Group E (Figure 3). A *V. metschnikovii* MLST database has not been established yet, and thus the *V. metschnikovii* genomes were not subject to these analyses.

### Comparison of Gene Functional Enrichment

**Figure 4:**
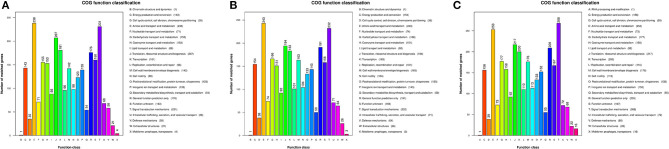
Functional classification of *V*. *cholerae* core genome COG functions. **(A)** COG functional classification of the core genomes of reference environmental *V*. *cholerae* strains; **(B)** COG functional classification of the core genomes of migratory bird *V*. *cholerae* strains; **(C)** COG functional classification of the core genomes of *V*. *cholerae* pandemic strains retrieved from the GenBank database.

Gene functions were predicted from the predicted genes and subjected to functional gene enrichment analysis. The *V*. *cholerae* core genes were enriched in numerous KEGG pathways including the “*E*,” amino acid transport and metabolism, and “*T*,” signal transduction mechanism pathways of the COG database. The numbers of genes within the “*T,”* signal transduction mechanism pathway, were enriched in group C genomes relative to “*E*,” amino acid transport and metabolism pathways. However, the other *V*. *cholerae* strain genomes exhibited opposite enrichment patterns (Figure 4).

To further investigate the functional differences encoded by the *Vibrio* genomes, we analyzed the “metabolism and environmental information processing” functional category encoded by different *V. cholerae* group genomes. Differences were observed in trimetlylamine oxide (TMAO) metabolism, in addition to two-component systems. Specifically, only group C genomes encoded complete TMAO systems. In addition, the genomes of the other groups lacked *TorA*, and these isolates are thus unable to metabolize TMAO. Differences between the two groups of *V. metschnikovii* strain genomes were minimal, although all group genomes were particularly enriched in genes involved in the “*E*,” amino acid transport and metabolism pathway.

### Pathogenicity and Antibiotic-Resistance Potential

The *V. cholerae* genomes generated in this study only encoded partial RTX protein structures and not *rtxA*. In addition, all of the groups encoded complete type VI secretory systems (T6SS). The presence of drug resistance genes for *V. cholerae* in the genomes generated here exhibited low frequencies of resistance genes and the lack of plasmids (Supplementary Table 4). All *V. metschnikovii* strains lacked CTX, and RTX. However, the genomes of the four strains from migratory birds (M13F, M9D, M29D, and M28D) encoded a T6SS system. Among these, three strains (M9D, M29D, and M28D) lacked other genes involved in secreting proteins (e.g., *hcp* and *vgrG*), while only M13F encoded a complete T6SS system *V. metschnikovii*.

## Discussion

*Vibrio cholerae* acquired cholera toxin (CTX), which is the primary reason for their ability to cause global pandemics, and CTX acquisition occurred over a long evolutionary history (39). CTX was likely introduced to *Vibrio* by the CTXϕ phage, while the toxin co-regulated pilus (TCP) is also a critical colonization factor of *V. cholerae* that serves as a receptor for CTXϕ (40). Other studies have indicated that AphA functions in a previously unknown step in the *ToxR* virulence cascade and helps activate the transcription of *tcpPH*. *TcpP/TcpH*, together with *ToxR/ToxS*, then activate *toxT* expression, ultimately resulting in the production of virulence factors like the cholera toxin and TCP (41–43). *Vibrio* strains isolated in this study exhibited unique combinations of virulence factor genes [e.g., *ctx*(-) *tcpA*(-) *hlyA*(+) *AphA*(+) *toxR*(+) *toxT*(-) *tcpP*(-) *tcpH*(-)], and thus they did not contain complete virulence factor regulatory networks based on the lack of *toxT*.

The group C genomes identified in this study also encoded the TMAO metabolic pathway, which differed from other *Vibrio* groups based on core-/pan-genome analysis. The TorR response regulator mediates TMAO induction of the *torCAD* operon expression and was intriguingly observed in all of the *V. cholerae* pandemic strains. With the exception of M66-2, all of the other strains in group C also encoded CTX. The original M66-2 strain did encode CTX, but CTX was knocked out of the strain in a previous study (44). The TMAO metabolic pathway exhibits a unique relationship with CTX production. Specifically, human intestines are anaerobic, and *V. cholerae*, as a facultative anaerobe, is able to grow by anaerobic respiration. CTX is a major virulence factor of *V*. *cholerae*, and its production is highly promoted during anaerobic growth using trimethylamine N-oxide (TMAO) as an alternative electron acceptor (45). The *V*. *cholerae* strains from environmental samples lacked the *torA* operon, which could lead to an inability to conduct TMAO respiration and thus produce CTX. However, the TMAO metabolic signaling pathway was not observed in the migratory bird *V*. *cholerae* strain genomes. Thus, it is likely that this metabolic pathway is not present in all *V*. *cholerae* strains. However, strain C16-2-29 shared very similar genomic attributes with the reference pandemic strains, unlike the other migratory bird *V. cholerae* strains. Specifically, the strain harbored the ability to encode a complete TMAO metabolic pathway, despite the apparent inability to produce CTX.

It should be noted that we were not able to exclude geographical factors that can influence bacterial evolution or differences in genomic contents. Strains that were isolated from the same host species but that exhibit differences in two-component systems will respond to external stresses differently. We speculated that conditionally pathogenic *V. cholerae* would unlike to evolve into a strain with CTX over a short period. Accordingly, the cholera pandemic strains have all been confirmed to be clonally related (2). The genomes of most strains belonging to group C contained fewer dispensable genes based on core-/pan-genomic analysis. A notable exception was the genome of TSY216, which contained more strain-specific genes. In addition to harboring two pairs of chromosomes, strain TSY216 also harbors a giant replicon that could explain its high strain-specificity relative to the others (46).

We additionally searched for other virulence genes in the genomes of *V. cholerae* from migratory birds by comparing them against the VFDB database. While the genomes contained some of the pathway components to produce the RTX toxin, they lacked the *rtxA* gene. *rtxA* deletion can cause a significant reduction in RTX toxin production (47). All strain genomes also encoded the T6SS system, while the *V. cholerae* genomes from migratory birds also harbored complete T6SS pathways based on KEGG analysis. T6SS was first discovered in *V. cholerae* in 2006 (48). Several of the *V. cholerae* groups encoded the T6SS system, and thus T6SS is probably a specific secretion system of *V. cholerae*. Only one of the three *V. metschnikovii* strain genomes contained the gene encoding the spike protein for T6SS. The *V. metschnikovii* strain encoding the spike protein of T6SS was isolated from migratory bird epidemic materials, while the *V. metschnikovii* strains that did not encode the spike protein of T6SS were isolated from healthy migratory bird feces in the second year. Importantly, T6SS without a spike protein does not confer pathogenic ability. Nevertheless, these results indicate that T6SS systems could have begun to evolve in *V. metschnikovii* strains. The M19X and M13F strains were closely related in the core genome phylogenetic analysis, although strain M13F did not encode a complete T6SS structure. Furthermore, strain M13F contained the HS I-I system that could activate T6SS, while M19X did not. Thus, different induction environments might activate the expression of HS I-I.

Phylogenetic analysis indicated that the *V. cholerae* strains from migratory birds (including strain NCTC30) were largely in group B, with the exception of C16-2-29. Strain NCTC30 was isolated from a World War One soldier with diarrhea (49). The strain harbored the T3SS-1 system, conferring it cytotoxicity (50). However, all of the migratory bird *V*. *cholerae* strains lacked the T3SS-1 system.

MLST analysis revealed a high degree of diversity among the strains that were evaluated. Indeed, these analyses indicated that the migratory bird strains harbored numerous new *V. cholerae* alleles and STs that have not been previously observed in databases. A total of seven different loci were used for MLST analysis of the environmental *V*. *cholerae* strains (excluding strain C16-2-29), while MLST analysis of the pandemic strains and C16-2-29 was performed using eight different loci (Supplementary Table 2). The combined phylogenetic analyses indicated that C16-2-29 was more closely related to the pandemic strains than to the strains isolated from the migratory birds.

In contrast to other migratory bird *V. cholerae* strains, C16-2-29 was classified within group A. The primary difference between the genome of this strain and other migratory bird *V*. *cholerae* strains was differences in the sequence of the Cbb3-type cytochrome oxidase. Accordingly, the genetic relationship between strain C16-2-29 and strains from group C was low. The COG group, “R,” functional genes were specifically enriched in the C16-2-29 strain relative to the others. In addition, C16-2-29 harbored a complete biosynthesis pathway for siderophore group non-ribosomal peptides based on KEGG annotations, while the other strains did not. The C16-2-29 strain was isolated from the Wuliangsuhai area of the Inner Mongolia autonomous region of China in 2017, while the other migratory bird *V. cholerae* strains were isolated from the Chifeng area of the Inner Mongolia autonomous region of China in 2018. Consequently, it is unclear whether differences in geographic location influence the evolution of *V. cholerae* migratory bird strain genomes.

Transmissible elements similar to plasmids were not found integrated into the *V. cholerae* and *V. metschnikovii* genomes from migratory bird strains, indicating little chance of transmitting and spreading resistance genes. Rather, inherent resistance mechanisms were identified on the genomic chromosomes. For example, efflux pump-based inhibition is likely to be a viable strategy to overcome antibiotic resistance for V. *cholerae* and *V. metschnikovii* strains present in migratory birds. An important pathogenic characteristic to identify is whether *V. cholerae* can form biofilms based on complete formation pathways. All of the strains analyzed here exhibited this capacity, except for the environmental strains and those from migratory birds. Moreover, the biofilm formation capacity of the *V. metschnikovii* strains was the same as the environmental strains and *V. cholerae* strains from the migratory birds, wherein the basement protein-forming gene *RmbA* was absent. Thus, biofilms formed by these strains might be thinner than those of group C, or they may not develop a three-dimensional structure (51, 52). In addition, CTXϕ phage has been observed in biofilms (53), although it is yet unclear whether a relationship exists between biofilm thickness and the presence of CTXϕ phage.

Overall, our study demonstrates that *V*. *cholerae* strains isolated from migratory birds do not exhibit genomic features consistent with an ability to cause pandemics, or otherwise be pathogenic. Specifically, these strains were identified as non-O1/O139 serotypes and only conditional pathogens. In addition, we document the first isolation of *V. metschnikovii* strains with complete T6SS pathways. Both *V*. *cholerae* and *V. metschnikovii* strains may cause intestinal discomfort in migratory birds through the activities of T6SS and hemolysin. In contrast, all strains with an ability to form biofilms were identified in aquatic plant or animal intestine environments.

## Data Availability Statement

The data presented in the study are deposited in the FigShare repository: https://doi.org/10.6084/m9.figshare.14417870.v2.

## Ethics Statement

All migratory bird stool samples were collected under the supervision by the Wild Animal Sources and Diseases Inspection Station, National Forestry and Grassland Bureau of China, and did not cause any harm to the animals. All migratory bird epidemic material samples were provided by the local animal disease prevention and control center for bacteriological examination. The experimental protocol was established, according to the ethical guidelines of Helsinki Declaration and was approved by the Laboratory Animal Welfare and Ethics Committee of the Institute of Military Veterinary Science, the Academy of Military Medical Sciences (AMMS - 11 - 2020 - 11).

## Author Contributions

PC and X-JG conceived, directed, and carried out the study. L-WZ, DC, L-HX, JJ, YS, and G-JL prepared samples for sequence analysis. XJ, J-yG, and LZ acquired samples and analyzed the data. All authors have read and approved the final manuscript.

## Conflict of Interest

The authors declare that the research was conducted in the absence of any commercial or financial relationships that could be construed as a potential conflict of interest.

## Abbreviations

PCR: Polymerase chain reaction
MLST: Multilocus sequence typing
ARDB: Antibiotic Resistance Genes Database
VFDB: virulence factor database
CARD: Comprehensive Antibiotic Research Database
COG: Cluster of Orthologous Groups of proteins
GO: Gene Ontology
KEGG: Kyoto Encyclopedia of Genes and Genomes
NR, Non-Redundant Protein Database. T6SS: Type VI secretory system
T3SS: Type III secretory system
CTX: cholera toxin.
